# Mending a broken heart: *In vitro*, *in vivo* and *in silico* models of congenital heart disease

**DOI:** 10.1242/dmm.047522

**Published:** 2021-03-28

**Authors:** Abdul Jalil Rufaihah, Ching Kit Chen, Choon Hwai Yap, Citra N. Z. Mattar

**Affiliations:** 1 Healthy Longevity Translational Research Programme, Yong Loo Lin School of Medicine, National University of Singapore, Singapore, 119228; 2 Department of Surgery, Yong Loo Lin School of Medicine, National University of Singapore, Singapore, 119228; 3 Department of Paediatrics, Yong Loo Lin School of Medicine, National University of Singapore, Singapore, 119228; 4 Division of Cardiology, Department of Paediatrics, Khoo Teck Puat -National University Children's Medical Institute, National University Health System, Singapore 119228; 5 Department of Bioengineering, Imperial College London, London, UK; 6 Experimental Fetal Medicine Group, Department of Obstetrics and Gynaecology, Yong Loo Lin School of Medicine, National University of Singapore, Singapore 119228; 7 Department of Obstetrics and Gynaecology, National University Health System, Singapore 119228

**Keywords:** Animal models, Biomechanics, Cardiogenesis, Congenital heart disease, Shear wall stresses, Structural anomalies

## Abstract

Birth defects contribute to ∼0.3% of global infant mortality in the first month of life, and congenital heart disease (CHD) is the most common birth defect among newborns worldwide. Despite the significant impact on human health, most treatments available for this heterogenous group of disorders are palliative at best. For this reason, the complex process of cardiogenesis, governed by multiple interlinked and dose-dependent pathways, is well investigated. Tissue, animal and, more recently, computerized models of the developing heart have facilitated important discoveries that are helping us to understand the genetic, epigenetic and mechanobiological contributors to CHD aetiology. In this Review, we discuss the strengths and limitations of different models of normal and abnormal cardiogenesis, ranging from single-cell systems and 3D cardiac organoids, to small and large animals and organ-level computational models. These investigative tools have revealed a diversity of pathogenic mechanisms that contribute to CHD, including genetic pathways, epigenetic regulators and shear wall stresses, paving the way for new strategies for screening and non-surgical treatment of CHD. As we discuss in this Review, one of the most-valuable advances in recent years has been the creation of highly personalized platforms with which to study individual diseases in clinically relevant settings.

## Abstract (precis)

**Summary:** *In vitro*, *in vivo* and *in silico* models of congenital heart disease provide important insights into the causes, evolution and pathological effects of congenital structural heart disease – a group of largely heterogenous disorders with substantial morbidity – and aid in designing effective therapies.

## INTRODUCTION

Congenital heart disease (CHD) refers to a heterogeneous collection of structural abnormalities of the heart or the great vessels. These are the most-common birth defect in newborns, affecting >1 million live births per annum globally and causing 10% of stillbirths, where moderate and severe forms affect 6–20 per thousand live-births (Hoffman and Kaplan, 2002). CHDs are a major cause of infant mortality and morbidity in the developed world (Fahed et al., 2013; Jin et al., 2017; Yang et al., 2006; Zaidi and Brueckner, 2017), accounting for ∼40% of infant deaths in North America (Agopian et al., 2017). Affected newborns, especially those with cyanotic CHD (Box 1, Glossary), may require palliative surgery during infancy, and affected children sometimes require subsequent invasive procedures and eventual heart transplants owing to cardiac failure caused by progressive ventricular dysfunction (Bacha et al., 2008; Yagi et al., 2018). Nevertheless, most CHD patients now are expected to survive, including children with complex cardiac defects (GB_2017_Congenital_Heart_Disease_Collaborators, 2020). This increased survival is attributable to innovative advances in imaging techniques, minimally invasive surgeries, improved clinical surveillance and translational research, all of which have dramatically improved the clinical management of CHD.

**Box 1. Glossary**

**Anisotropic hyperelastic constitutive law:** a constitutive law is a mathematical description of the mechanical properties of matter. The hyperelastic model is a form of constitutive law that captures elastic properties well. Anisotropic refers to a material that has different mechanical properties (such as stiffness) in different directions, and is opposed to isotropic, which refers to material that has the same properties in any direction.

**Coarctation of the aorta** **(CoA):** narrowing of the aorta between upper and lower body branches, typically just after the arch of the aorta.

**Aortic isthmus:** the section of the aorta between its connection to the left subclavian artery and the ductus arteriosus.

**Aortic valvuloplast**y**:** a surgical procedure to expand the stenosis at the aortic valve by using a percutaneous balloon catheter.

**Cardia bifida:** an embryonic malformation incompatible with life, denoting a ‘double’ or ‘split’ heart in which heart tube formation produces two heart cylinders instead of one, arresting normal development and circulation. It arises from improper cell migration or differentiation and is often genetically driven.

**Cardiac neural crest:** multipotent embryonic cells that undergo epithelial-mesenchymal transition and migrate to the heart via pharyngeal arches 3, 4 and 6. The cardiac neural crest complex aids cardiac outflow tract and aortic arch modeling.

**Chromatin quantitative trait loci (chQTL):** DNA loci that encode a quantitative phenotypic trait of an organism, which is identified in the genome by the association of certain molecular signatures (e.g. single-nucleotide polymorphisms) with the observed physical characteristic(s). These loci underlie characteristics that can be described as continuous, e.g. a severity spectrum of disease phenotype, which may be polygenic and under environmental influence, rather than being a discrete (e.g. present/absent) trait.

**Conotruncal defects:** this group of CHD affects the cardiac outflow tracts (pulmonary artery and aorta) and is frequently associated with the 22q11.2 deletion (DiGeorge syndrome). Examples include pulmonary atresia, double-outlet right ventricle, double-outlet left ventricle, transposition of the great arteries and tetralogy of Fallot.

**Critical regulators of organ developmen**t**:** proteins that fulfil fundamental roles in cardiac and other organ development, an example of which are the NKX-homeodomain factors (see below).

**Cyanotic congenital heart disease:** a group of CHD that cause chronically low blood oxygenation, leading to a blue (cyanosed) appearance of the lips, fingernails and skin.

**Double-outlet left ventricle (DOLV)/double-outlet right ventricle (DORV):** rare cardiac malformation in which both great arteries originate from the morphological left/right ventricle.

**Doppler optical coherence tomography:** non-invasive imaging technique that uses backscattered light and Doppler waveforms to achieve high-resolution tomographic images of biological tissues. It is particularly effective for *in vivo* blood flow imaging.

**Ductus venosus (DV):** a vein that shunts a portion of umbilical vein blood flow directly to the inferior vena cava in a fetus.

**E- and A-waves:** two peaks in the cardiac ventricular inflow velocity waveforms during diastole, when the heart muscle relaxes. E-wave corresponds to first wave of inflow caused by the passive relaxation of the ventricle, whereas A-wave corresponds to the second wave of inflow caused by active contraction of the atrium.

**Fetoscopy:** surgical procedure that uses a small-calibre laparoscope to obtain minimally invasive access to the fetus, amniotic cavity, umbilical cord and the fetal side of the placenta.

**First and second heart fields:** the heart fields arise from the embryonic mesoderm; the primary (first) heart field forms the initial heart tube, left ventricle and left and right atria, whereas the second heart field contributes to the right ventricle, both atria and the outflow tracts.

**Fontan surgery:** surgical repair of single-ventricle congenital heart malformations that leads to systemic flow of venous blood to the lungs without passing through a ventricle.

**Heterotaxy syndrom**e**:** a series of congenital defects caused by disruption of left-right laterality, i.e. the programmed rotation of organs so that they reach their final location within the body cavity, resulting in disordered arrangement of thoracic and abdominal viscera in relation to the body axis.

**His–Purkinje system:** fibers located in the atrial and ventricular myocardium that form the electrical conduction pathway of the heart. The bundle of His transmits signals received from the sinoatrial and atrioventricular nodes to the Purkinje fibers. The latter split in the interventricular septum to supply both ventricles with electrical signals that initiate contractions.

**Mitral and tricuspid valves:** atrioventricular valves control the direction of blood flow from the atrium into the ventricle. The mitral valve is in the left heart and the tricuspid valve in the right heart.

**Navier–Stokes equations:** a set of equations that describe the physics of fluid flow, relating fluid flow patterns to fluid forces, derived from the conservation law principles of mass, momentum and energy.

**Nkx5-2:** homeodomain transcription factors, which exhibit a characteristic folded structure that allows them to bind target DNA to regulate gene expression. These transcription factors are important for cell differentiation during the early embryonic period and, thus, essential in cardiogenesis. Mutations cause disorders of normal heart development.

**Proepicardium:** transient extracardiac tissue that appears in the embryonic period and aids cardiogenesis in vertebrates. It arises from the lateral plate mesoderm. Mesothelial cells cross from the proepicardium to attach to the myocardium, eventually forming the epicardium.

**Pulmonary atresia:** a condition in which the pulmonary valve develops incorrectly, prohibiting blood flow between the right ventricle into the lungs.

**Sarcomere:** a unit of striated muscle, whose long fibrous protein filaments slide past each other during muscle contraction or relaxation, composed of actin (thin filament, bound to the Z lines that define the sarcomere unit) and myosin (thick filament, bound to adenosine triphosphate providing energy for action).

**Short-hairpin RNAs (shRNAs):** artificial RNA molecules that comprise a tight hairpin turn to silence endogenous messenger RNA translation via the RNA-induced silencing complex that binds to and cleaves mRNA or suppresses its translation. Expression of shRNA in the target cell is accomplished by using viral vectors or plasmids.

**Slit-Robo:** a cellular signaling complex involved in axon development and branching, as well as neuronal cell migration. The complex comprises the secreted protein Slit and its transmembrane receptor Roundabout (Robo).

**Spatio-temporal image correlation (STIC**)**:** a mode of ultrasound imaging providing 4D B-mode images (3D over time), enabled by image-correlation processing and a slow motorized out-of-plane sweep of 2D scanning elements during the scan.

**Tetralogy of Fallot (TOF):** a common cyanotic CHD, characterized by pulmonary stenosis/right ventricular outflow tract obstruction (RVOTO), ventricular septal defect (VSD), overriding aorta and hypertrophy of the right ventricle (RVH).

**Transposition of the great arteries:** rare heart defect due to a positional swap of the pulmonary artery and the aorta. Transposition of the great arteries: discordant ventriculo-arterial connection – the right ventricle is connected to the aorta (instead of pulmonary artery), and left ventricle to pulmonary artery (instead of aorta).

CoA, coarctation of the aorta; ECM, extracellular matrix; EMT, epithelial to mesenchymal transition; HLHS, hypoplastic left heart syndrome; LPA, left pulmonary artery; TOF, tetralogy of Fallot.

**Fig. 1.**
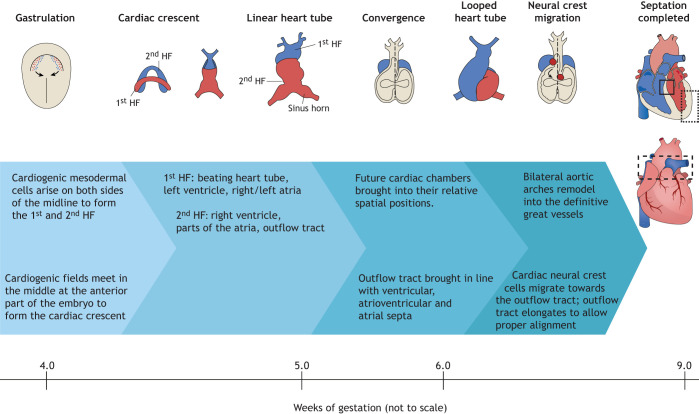
**Cardiac development in the human embryo.** This schematic shows the embryonic development of the human heart through first and second heart field (HF) formation, heart tube formation and pumping, looping, neural crest migration and septation, resulting in a fully developed heart at the end of gestation. Boxed areas highlight structures typically affected by congenital malformations (solid lines: atrial and ventricular septal defects; dotted lines: hypoplastic ventricles; dashed lines: aortic and pulmonary valve defects and defects of the great vessels, such as transposition).

CHD is caused by abnormal cardiogenesis, a complex developmental process (Fig. 1) governed by multiple interlinked and dose-dependent pathways (Witman et al., 2020). Although 80–85% of CHDs are caused by multifactorial or unidentified causes, large epidemiological and molecular studies have identified monogenic (3–5%) or chromosomal (8–10%) anomalies, copy number variants (3–25%) and environmental causes (2%), such as maternal diabetes, smoking or alcohol use, in ∼20–30% of patients (Cowan and Ware, 2015; Hoffman and Kaplan, 2002; Kuciene and Dulskiene, 2008; Meberg et al., 2007; van der Bom et al., 2011). The genetics of CHD are also complex; a single candidate gene or genetic variant can produce a spectrum of heart malformations and may even occur in phenotypically normal humans (Table 1). Variance in genetic penetrance also occurs within affected families, resulting in a range of CHD phenotypes. These events increase the difficulty of identifying and characterizing the genetic risk factors for CHD, and emphasize the importance of understanding cardiogenesis at the molecular level (Brown et al., 2004; Farr et al., 2018).

**Table 1.**
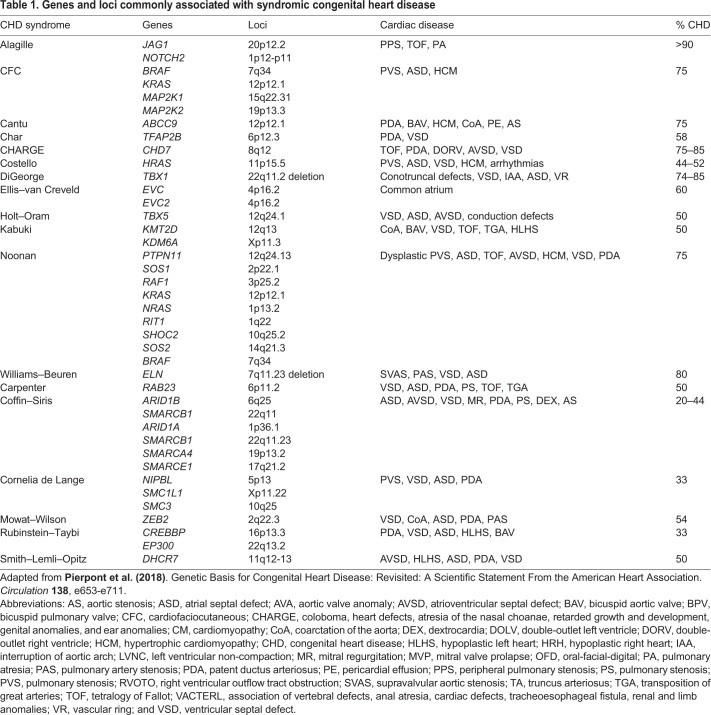
**Genes and loci commonly associated with syndromic congenital heart disease**

There remains much to learn about the underlying causative mechanisms of CHD, which we are only now beginning to understand through tissue and computational modeling. As a result, existing clinical therapies remain palliative at best as they do not address the underlying mechanisms and causes of CHD. Among the most-influential genetic factors that contribute to CHD are multiple under- or over-expressed genes, incomplete penetrance of variants, aberrant epigenetic controls of gene expression and mechanobiological effects on the developing heart structures.

Here, we review the tissue and computational models that have enabled researchers to uncover several associated and causative factors implicated in various CHD. We aim to provide scientists and clinicians with an overview of the abnormal cardiogenic pathways uncovered by studying single cells, organoids, animal models and, intriguingly, *in silico* computational models of normal and abnormal human fetal hearts. We discuss how these have led to a better understanding of the impact of genetic or physical insults has on the developing fetal heart, particularly for readers interested in CHD research. The incidence of CHD in adults is increasing steadily with advances in cardiac interventions (Pierpont et al., 2018), which have reduced CHD-related infant mortality, highlighting the pressing need for molecular therapies that address actual causes rather than physical consequences and that can be implemented early in development, such as in the young infant or even the developing fetus.

### An overview of CHD

**Fig. 2.**
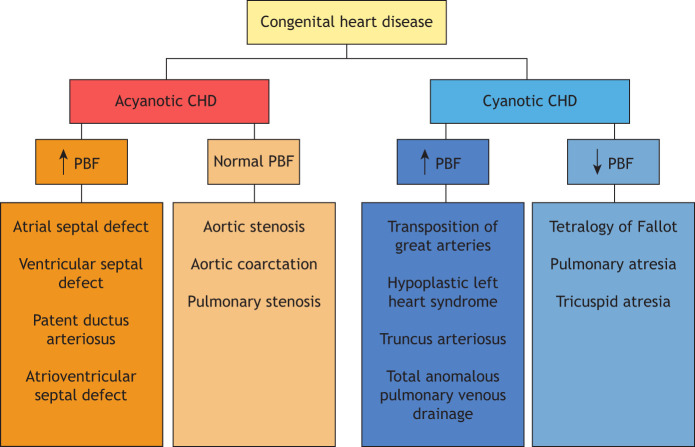
**Classification of CHD into acyanotic and cyanotic CHD, and the status of pulmonary blood flow (PBF).**

As CHD comprise a heterogeneous group of cardiac malformations, the key clinical manifestation depends on the type of CHD. Classification schemes have evolved substantially since Maude Abbott generated *The Atlas of Congenital Cardiac Disease* more than 100 years ago (Abbott, 1936). Broadly speaking, CHD can be classified, based on morphology and hemodynamics, into cyanotic and acyanotic CHD (Fig. 2). Cyanosis is a bluish discoloration of the skin and mucous membrane, resulting from reduced oxygen saturation of the circulating blood. Patients with cyanotic CHD have mix of deoxygenated and oxygenated blood, with an overall reduction of circulating oxyhemoglobin.

Tetralogy of Fallot (TOF) is a common cyanotic CHD characterized by pulmonary stenosis, right ventricular outflow tract obstruction (RVOTO), ventricular septal defect (VSD), overriding aorta and right ventricular hypertrophy (Lahm et al., 2015). Owing to the RVOTO, deoxygenated blood in the right ventricle shunts across the VSD into the left ventricle and out through the aorta to enter systemic circulation, resulting in reduction of systemic oxygen saturation and, consequently, cyanosis. Another example of cyanotic CHD is hypoplastic left heart syndrome (HLHS), a critical CHD characterized by underdevelopment of the left-sided structures of the heart, including the ascending aorta, left ventricle and aortic and mitral valves (Box 1), often culminating in childhood death (Barron et al., 2009; Tchervenkov et al., 2006). HLHS results from diminished ventricular flow and disruption of genetic regulatory networks governing left ventricular chamber development (deAlmeida et al., 2007; Hinton et al., 2007; Iascone et al., 2012).

Common examples of acyanotic CHD include atrial septal defect (ASD) and VSD. In ASD, there is a hole in the septum, the wall that divides the left and right atrium; in VSD, the defect is in the septum between the left and right ventricle. In both defects, oxygenated blood shunts from the oxygen-rich (left) chambers to the oxygen-poor (right) chambers; therefore, these lesions are also termed left-to-right shunts. As the mixing is from the oxygenated to the deoxygenated chambers, there is no reduction in the oxygen saturation in the systemic circulation and, thus, no cyanosis.

The etiology of CHD is multifactorial, with only 30% of cases traced to a known cause (Pierpont et al., 2018). Causes of CHD are divided into genetic (Table 1) and non-genetic. Non-genetic etiologies of CHD such as environmental teratogens, infectious agents, and maternal diabetes mellitus have been widely studied (Fahed et al., 2013; Zhu et al., 2009) but there is still much to be uncovered regarding the genetics and epigenetics of CHD (Fahed et al., 2013; Zhu et al., 2009). Epidemiology points to a strong genetic contribution regarding CHD, including a higher concordance of CHD among monozygotic compared to dizygotic twins, and an elevated recurrence risk of related forms of CHD among siblings (Lyu et al., 2018; Pediatric Cardiac Genomics et al., 2013; Zaidi and Brueckner, 2017). By contrast, there is no family history of CHD for a large proportion of particularly severely affected patients (Burn et al., 1998), which implies that *de novo* genetic events significantly contribute to the etiology of CHD, including chromosomal abnormalities, point mutations and structural and copy number variants (Zaidi et al., 2013). It is now estimated that ≤35% of CHD cases, with or without extracardiac anomalies, can be attributed to genetic factors, including copy number variation (Breckpot et al., 2010; Goldmuntz et al., 2011; Lalani et al., 2013; Richards et al., 2008; Syrmou et al., 2013; Thienpont et al., 2007), chromosomal abnormalities (Roos-Hesselink et al., 2005; van der Bom et al., 2011) or a single gene mutation (van der Bom et al., 2011).

### *In vitro* modeling of CHD

Evidence accumulated over the past decade demonstrates the power of human induced pluripotent stem cell (iPSC)-based disease modeling as investigative tools for biomedical research and regenerative medicine. iPSCs may, to various degrees, recapitulate the pathophysiological and clinical manifestations of human disease. Additionally, they can be derived from any individual, thereby, providing the ability of unlimited supply of cells bearing clinically relevant phenotypes, presenting unprecedented opportunities to evaluate the etiology and progression of a variety of inherited cardiac diseases.

### iPSC-derived models of CHD

The controlled differentiation of iPSCs into cardiomyocytes provides a unique platform with which to establish personalized models of CHD that feature patient-specific factors and phenotypes. As originally reported in 2007, overexpression of the transcription factors Oct3/4 (officially known as *POU5F1*)*, Sox2, Klf4* and *Myc* (also known as Yamanaka factors) can induce human fibroblasts to become pluripotent (Takahashi et al., 2007). Since this ground-breaking study was first published, a plethora of reprogramming cocktails have been designed to convert various somatic cells to pluripotency (Lee et al., 2010; Liao et al., 2008; Maherali et al., 2008; Okita et al., 2013). The key advantages of iPSCs are (1) derivation from human somatic cells, which in some jurisdictions would more readily receive ethical approval for generation and use (Bilic and Izpisua Belmonte, 2012); and (2) preservation of the donor's genomic and epigenetic profile to study precise genetic mutations (Parrotta et al., 2020). Here, we review the use and potential applications of iPSC-derived cardiac models for investigating the pathophysiology of complex CHD and designing novel treatment strategies.

For successful generation of CHD models from iPSCs, it is crucially important to adopt protocols that can induce, expand and purify functional cardiomyocytes. Substantial progress has been made in recent years towards understanding the regulatory mechanisms of cardiac differentiation, and in developing robust strategies to generate iPSC-derived cardiomyocytes efficiently and reproducibly. These differentiation protocols often recapitulate the multi-stage developmental processes of mesoderm commitment and cardiac specification; some involve spontaneous differentiation from embryoid body systems (Kattman et al., 2011; Mummery et al., 2012), whereas others employ adherent monolayer culture systems (Kadari et al., 2015) and chemically defined serum-free cultures (Burridge et al., 2014; van den Berg et al., 2016). These different protocols, nevertheless, typically feature a core set of regulatory networks and signaling pathways that play essential roles in establishing the cardiovascular system, including bone morphogenic proteins (BMPs), fibroblast growth factors (FGFs) and WNT signaling factors (Kattman et al., 2011; Lian et al., 2012; Tirosh-Finkel et al., 2010; Zhao et al., 2019). The iPSC-derived cardiomyocytes generated according to these different protocols typically beat spontaneously, express channels and sarcomeric components, and exhibit Ca^2+^ transients and action potentials (Karakikes et al., 2015; Ma et al., 2011; Zhang et al., 2009; Zwi et al., 2009).

### Hypoplastic left heart syndrome

**Fig. 3.**
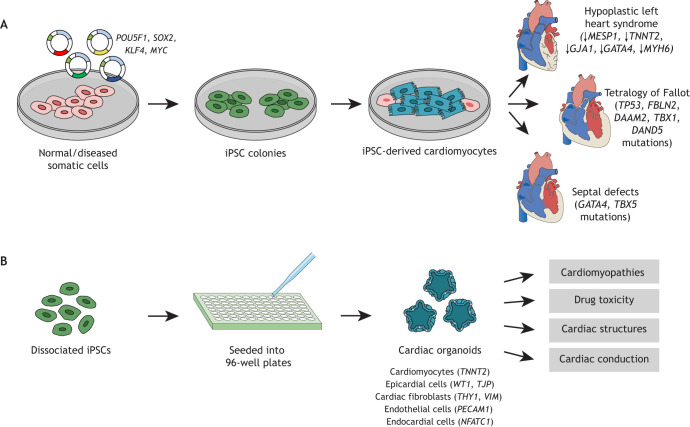
***In vitro* modeling of CHD.** (A) Patient-derived fibroblasts or other somatic cells are induced into pluripotent stem cells (iPSC) by addition of the Yamanaka factors POU5F1, SOX2, KLF4 and MYC. The resulting iPSCs are subsequently reprogrammed in differentiation medium containing differentiation and growth factors, such as human BMP4, activin, Wnt/β-catenin and FGF to yield cardiomyocytes bearing the gene defects of interest; i.e. reduced gene expression (HLHS), mutations (TOF, septal defects). (B) The resulting individual iPSCs can be used to study the cellular and molecular signatures in 2D cell structures relevant in a number of CHDs or can be further cultured in 3D to form cardiac organoids to study other environmental interactions. TNNT2, cardiac troponin 2; WT1, WT1 transcription factor (Wilms tumor 1); TJP, tight junction proteins 1, 2 and 3; THY1, Thy-1 cell surface antigen, VIM, vimentin; PECAM1, platelet and endothelial cell adhesion molecule 1; NFATC1, nuclear factor of activated T cells 1 (cytoplasmic).

As discussed earlier, HLHS (Figs 1 and 3A) is a common and serious congenital heart defect that often results in childhood death (Barron et al., 2009; Tchervenkov et al., 2006). Cardiomyocytes have been produced from iPSC lines derived from patients with HLHS, albeit with lesser efficiency than iPSC production from normal donors (Jiang et al., 2014; Kobayashi et al., 2014). Cardiomyocytes generated from HLHS-derived iPSCs showed developmental and functional defects, and more-primitive cardiac phenotypes in gene expression studies compared to control-iPSC lines and embryonic stem cells. The cells demonstrated reduced expression of several key cardiac mesoderm marker genes, including those of mesoderm posterior BHLH transcription factor 1 (*MESP1*), cardiac troponin T2 (*TNNT2*) and connexin 43 (*GJA1*), which mediates electromechanical transduction and cardiomyocyte alignment, and delayed expression of cardiac progenitor marker gene GATA binding protein 4 (*GATA4*) during cardiac differentiation. HLHS-derived cardiomyocytes also showed accelerated decrease of Ca^2+^ and generated Ca^2+^ transients in the presence of caffeine, indicating dysfunctional ryanodine receptors, huge ion channels that mediate Ca^2+^ release from the sarco/endoplasmic reticulum into the cytoplasm (Jiang et al., 2014; Kobayashi et al., 2014). These findings indicate that HLHS patient-derived cardiomyocytes undergo impaired cardiac lineage differentiation, involving cardiac mesoderm formation, cardiac progenitor maturation and cell commitment to atrial or ventricular phenotypes. A new mechanism for HLHS development has also been discovered in studies that used iPSC, and potentially involves a variant of myosin heavy chain, cardiac muscle alpha isoform (*MYH6*), i.e. *MYH6*-R443P, which results in an arginine to proline change in MYH6 (Kim et al., 2020). In this study, iPSC-derived cardiomyocytes were generated from a HLHS-affected family comprising one affected proband and one affected and one unaffected parent. Resulting cardiomyocytes showed dysmorphic sarcomeres (Box 1) that caused decreased atrial contractility and compensatory increased expression of *MYH7*. When the ‘phenotypically normal’ parental iPSCs were edited by using CRISPR/Cas9 to contain the *MYH6*-R443P variant, the resulting cardiomyocytes reproduced the HLHS phenotype of the proband. By contrast, correction of the *MYH6*-R443P variant to wild-type *MYH6* by using gene editing in iPSCs of the proband rescued cardiomyogenic differentiation, contractility and velocity, and sarcomere organization. This is the first report describing atrial sarcomere disorganization in cardiac tissues from HLHS patients with *MYH6* variants to lead to decreased atrial contractility and, consequently, hypoplastic left ventricular development. Although not unexpected – since *MYH6* is more abundantly expressed in atria than in ventricles – these findings demonstrate the feasibility of using HLHS-derived iPSC-cardiomyocytes as *in vitro* tools to test drugs that target atrial contractility to alleviate HLHS deficiencies, or to design strategies that alter developmental pathways in order to minimize or even prevent development of *MYH6*-variant-associated HLHS.

### Tetralogy of Fallot

As described above, TOF (see Figs 1 and 3A) causes a severe cyanotic form of CHD (Lahm et al., 2015). In 2020, Grunert et al. generated iPSCs from two well-characterized TOF patients and differentiated them into cardiomyocytes by using standard methods (Grunert et al., 2020). The authors described using TOF-derived iPSC-cardiomyocytes for the first time as an *in vitro* tool to study the transcriptional landscape of TOF. Some of their findings included disease-causing mutations in tumor protein p53 (TP53), which is important for smooth muscle cell migration, fibulin 2 (FBLN2), which regulates atrioventricular valvular-septal morphogenesis, and in dishevelled associated activator of morphogenesis 2 (DAAM2), which mediates sarcomere assembly and myocardial maturation (Grunert et al., 2020). In a second study, Han et al. generated iPSCs from a child diagnosed with TOF, who carried a mutation in T-box transcription factor 1 (TBX1) (Han et al., 2020). TBX1 is known to be involved in cardiac outflow tract formation, demonstrating the contribution of the TBX1 mutation to TOF-associated conotruncal defects (Box 1) (Hasten et al., 2018). Such patient-specific iPSC lines are useful tools for proving that a genetic mutation causes the observed phenotypes and for investigating gene therapy options for TOF. For example, homozygous correction of the c.455G>A mutation in the *DAND5* gene has been achieved by using CRISPR/Cas9 (Inácio et al., 2020). DAND5 is involved in the NODAL signaling pathway that controls early embryo patterning, mesoderm and endoderm formation, and the specification of left-right asymmetry (Barnes and Black, 2016).

### Septal defects

VSD and ASD account for almost 50% of all CHD (Hoffman, 1995). In mice, deletion of *Gata4* results in cardia bifida (Box 1) and early fetal death (Misra et al., 2012). In humans, the heterozygous missense mutation G296S in the cardiac transcription factor *GATA4* causes ASD, VSD and pulmonary valve stenosis (Garg et al., 2003). *In vitro* investigations demonstrated that this *GATA4*-G296S mutation reduces DNA-binding affinity and transcriptional activity of the resulting GATA4 mutant protein, and abolishes its interaction with T-box transcription factor 5 (TBX5), which is important for normal heart formation (Ang et al., 2016; Garg et al., 2003). Cardiomyocytes derived from iPSCs of patients harboring *GATA4* mutations also show disruption of interactions between transcriptional proteins; an example is the loss of TBX5 recruitment to the cardiac super-enhancers (elements that are associated with genes for heart development and muscle contraction). This leads to impaired cardiomyocyte contractility, Ca^2+^ handling and metabolic activity (Ang et al., 2016). Taken together, these studies confirm the crucial role of *GATA4* and *TBX5* in maintaining the robust cardiac gene programming necessary for the prevention of septal defects in both murine and human cardiovascular systems (CVSs).

### Organoids and engineered 3D models

Despite the importance of traditional 2D models, in particular their cost-effectiveness, reproducibility and complementarity to animal models, their ability to recapitulate *in vivo* cardiac architecture and simulate cardiac tissue complexity and organization is limited. Cell-cell interactions are restricted to side-by-side contact. This limitation, potentially, affects cellular morphology, survival and proliferation, which in turn restricts the study of disease mechanisms in such 2D settings. To circumvent the intrinsic limitations of conventional 2D cell cultures, human cardiac organoids have been generated using 3D culture systems, which can more closely recapitulate endogenous cardiomyocyte tissues by incorporating key elements of the cardiac system, including supporting scaffolds and external stimuli (Fig. 3B). Thus, cardiac organoids are, potentially, useful for investigating human heart development and the etiology of CHD, as they facilitate physiologically relevant experiments that would be ethically or technically impossible to perform *in vivo*. 3D cardiac organoids also provide more-robust biological models for studying tissue regeneration and performing drug toxicity screens.

Studies have shown that human iPSC-derived cardiomyocytes cultured in 3D undergo greater maturation, exhibit improved adult cardiomyocyte physiology, and show increased contractility and electrical function relative to cardiomyocytes differentiated and cultured in 2D (Correia et al., 2018; Karbassi et al., 2020; Lemoine et al., 2017). 3D cardiac organoids derived from human iPSCs also enabled modeling of early heart field development (Andersen et al., 2018) and aided identification of signaling pathways involved in the development of both first and second heart fields (Box 1) (Kelly et al., 2014), furthering our understanding of heart field evolution and chamber-specific cardiac pathogenesis (Andersen et al., 2018).

Cardiac organoids can be generated using a highly efficient, scalable and reproducible method (Israeli et al., 2020 preprint), and can be differentiated from several human iPSC lines by using stepwise chemical modulation of the Wnt signaling pathway under completely defined culture conditions. The resulting organoids start to contract around day 6 of culture and grow up to 1mm in diameter by day 15 post differentiation. Cardiac organoids consist of multiple cell types, and can exhibit interconnected internal chambers and morphological complexity similar to that of the human fetal heart (Israeli et al., 2020 preprint). The main cardiac-related lineages contained within these organoids, as exhibited on confocal microscopy and cell marker analyses, include cardiomyocytes (*TNNT2*), epicardial cells (*WT1*, *TJP*), cardiac fibroblasts (*THY1*, *VIM*), endothelial cells (*PECAM1*) and endocardial cells (*NFATC1*). Provided in parenthesis are the marker genes generally used to isolate or identify the cells of interest (Israeli et al., 2020 preprint).

The ability to evaluate contractile function and Ca^2+^ handling of cardiomyocytes is highly relevant for studying the most common cause of cardiac death in CHD patients – chronic heart failure. To recapitulate more closely the endogenous architecture and mechanics of cardiomyocytes, 3D-engineered heart tissues (EHT) have been developed. 3D EHTs are generally constructed using both cardiomyocytes and non-cardiomyocytes, such as fibroblasts and endothelial cells, mixed with biodegradable matrices that can be polymerized into sheets, cylinders or rings. With cyclic strain and electrical pacing in 3D culture, these cells first remodel the extracellular matrix (ECM), over time forming a structure resembling the intact myocardium with uniformly aligned cardiomyocytes that form gap junctions. The EHTs generally remain viable in culture for 4–6 weeks without overgrowth of non-cardiomyocytes or loss of contractile performance; various testing apparatuses can be used to measure Ca^2+^ transients, action potentials and twitch forces (Hirt et al., 2014). EHTs are also constructed by casting hydrogel-containing cardiomyocytes into moulds maintained under a mechanically defined load. Subsequently, cardiomyocytes in the hydrogel remodel and align relative to the mechanical load to form coherently beating syncytia (Lee et al., 2017). This configuration allows the analysis of contractile force under stable conditions that simulate the cardiac microenvironment, i.e. 3D heart-like muscle strips that contract under auxotonic conditions (Mannhardt et al., 2016). Mature 3D EHTs have been fabricated from iPSCs in a custom-designed bioreactor that generates electromechanical stimulation (Ronaldson-Bouchard et al., 2019), an attractive system with great potential for translational research, enabling assessment of cardiac contractility, and facilitating investigation of various genetic mutations and molecular mechanisms of CHD-related heart failure. Recently, by using single-cell RNA sequencing (scRNAseq), 2D and 3D cardiac tissues served as powerful tools with which to understand the transcriptomic basis and molecular regulation of iPSC-cardiomyocytes that carry disease-associated genes, further expanding the repertoire of CHD research (Lam et al., 2020; Ni et al., 2020).

### Challenges

Patient-derived iPSCs and iPSC-derived cardiomyocytes have well-established advantages as models of cardiac disease, given that they recapitulate the pathophysiological characteristics of a broad range of CHDs (Ebert et al., 2012; Parrotta et al., 2020). However, it is uncertain how closely these *in vitro* iPSCs and iPSC-derived cardiomyocytes recapitulate the disease phenotype of the donor, particularly its severity. *In vitro* differentiated cardiomyocytes have, typically, immature structures and function (Karakikes et al., 2015; Veerman et al., 2015). Their gene expression profiles resemble those of fetal cardiomyocytes, with smaller resting membrane potentials and upstroke velocities, relatively fewer mitochondria, and shorter and more disorganized sarcomeres relative to those of adult cardiomyocytes (Gherghiceanu et al., 2011; Karakikes et al., 2015; Koivumäki et al., 2018; Veerman et al., 2015; Yang et al., 2014). Incomplete pluripotency reprogramming and errors that occur during reprogramming can also alter phenotype and function. Atypical methylation patterns and genetic mutations in iPSCs are both associated with variants in parental somatic cells, with reprogramming or with the duration of *in vitro* culture (Yoshihara et al., 2017). Additionally, the various differentiation protocols used can result in iPSC-derived cardiomyocytes, with heterogenous subtypes resembling atrial, ventricular and nodal cells (Burridge et al., 2015). With varying ratios of cardiomyocyte subtypes, the comparability of readouts, like contractile force and electrophysiological activity, may be compromised (Streckfuss-Bömeke et al., 2013; van den Heuvel et al., 2014). Another limitation of iPSC-generated disease models is their inability to fully recapitulate a clinical condition *in vitro*, often due to absence of the complicated interplay among multiple genetic and environmental factors, including age, sex and ethnicity. It is important not to overlook these relative disadvantages when interpreting data from iPSC-derived cardiomyocytes. This discussion, reviewed in detail elsewhere (Doss and Sachinidis, 2019) highlights the importance of ongoing strategies in developing authentic health and disease models, and their complementary bioassays for the functional testing of drugs and other therapeutic interventions.

### *In vivo* modeling of CHD

Animal models, large and small, have been instrumental in the discovery of the multigenic nature of CHD and its complex cardiac morphologies (Kraft and de Andrade, 2003). Large, conserved networks of genes control early cardiogenesis and developing cardiac function, and many are highly conserved in lower-order animals that can be genetically manipulated, bred and studied with relative ease (Bier and Bodmer, 2004). With a range of widely available molecular techniques, such as morpholino-oligonucleotides (MOs) and gene editing techniques (reviewed by Kaltenbrun et al., 2011; Zhang and Liu, 2015), candidate genes can be knocked down, knocked out or knocked in, enabling studies of their causality and supporting genetic screens. Higher-order animals have, as yet, not been used to model CHD due to the technical difficulties, time and expense involved in breeding, for example, transgenic sheep or non-human primates through assisted reproductive programmes, and due to the lack of naturally occurring structural anomalies that resemble human CHD (Moran et al., 2015; Proudfoot et al., 2015). Nevertheless, large animals are valuable as surgical models of outflow track obstruction, complex vascular configurations that mimic TOF, and aortic or pulmonary stenosis – both of which can be produced *in utero* (Table 2).

**Table 2.**
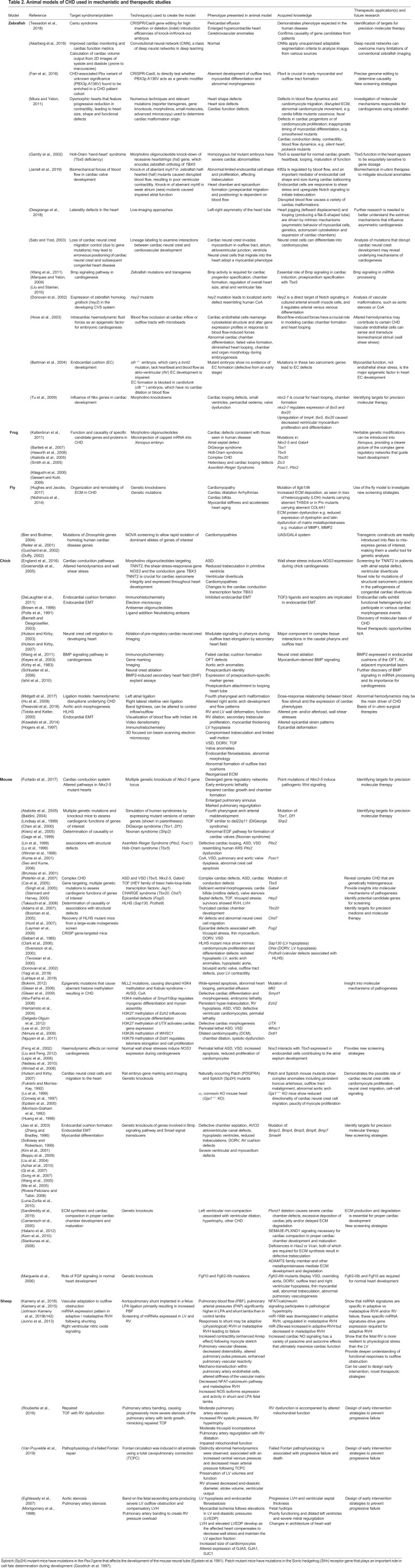
**Animal models of CHD used in mechanistic and therapeutic studies**

The main advantage of using lower-order animal species to model CHD are their low cost, fast reproduction times and genetic tractability, made by possible by the fact that the genetic and epigenetic elements that contribute to human CHD are highly conserved in fly, zebrafish, frog, chicken and mouse (Bakkers, 2011; Bier and Bodmer, 2004; Gut et al., 2017; Kooij et al., 2014; Sparrow et al., 2000). These models were used to great effect to investigate the impact of genes and gene dosing (via transgenesis), and of silencing (via MOs) on the aetiology and pathology of CHD. They were also used to investigate new candidate genes that had been detected through whole-genome sequencing of patient samples and then introduced into animal genomes by using precise molecular tools (Kaltenbrun et al., 2011). Together, these models helped to uncover the diverse genetic and non-genetic mechanisms that regulate cardiovascular physiological and pathological processes, ECM remodeling and mechanobiology (Table 2). In this section, we discuss the advantages and limitations of each animal model of CHD, as well as the genetic screening and corrective strategies that rely on a certainty of causation – which can be difficult to prove in human patients.

### Zebrafish

Although the zebrafish (*Danio rerio*) heart develops into a simple, single ventricular structure, its cardiac function is similar to that of the human CVS. In addition, heart development in zebrafish relies on the same proteins as does heart development in humans (Farr et al., 2018; Khodiyar et al., 2013) and can also be altered by certain environmental toxins (reviewed by Brown et al., 2016). The heart rate and electrophysiological cardiac behavior of zebrafish are similar to these parameters in human (Genge et al., 2016; Sedmera et al., 2003). Genetic pathways influential in cardiogenesis are also highly conserved between humans and zebrafish, and specific molecular and cellular tools are available to investigate their function, including MOs, gene-editing tools and cardiac-specific fluorescent transgenes that can be used to investigate cardiovascular pathology in individual heart structures (Gut et al., 2017). Zebrafish embryos are also fertilized externally and are transparent, facilitating their real-time live imaging using high-speed fluorescent microscopy. However, the simple configuration of zebrafish hearts makes studying the structurally complex CHD abnormalities challenging, as the phenotypes caused by CHD-associated genetic mutations might be quite different compared with their human counterparts (Poon and Brand, 2013). Other species-specific differences include the absence of a His-Purkinje system (Box 1) (Sedmera et al., 2003), and genetic redundancy, which can hamper gene dosage studies (Peng, 2019). In addition, there is the challenge of accurately measuring cardiac volume and output, which are often prone to bias, from 2D zebrafish images (Akerberg et al., 2019). Given these limitations, zebrafish is best used to screen for novel drug therapies rather than to assess genetic, cell or surgical therapies, which limits the translational applicability of this model.

### Frog

The African clawed frog (*Xenopus laevis)* has contributed greatly to our understanding of vertebrate development and gene function, with the availability of established molecular tools with which to investigate, by gene knock-in or knockdown, the functions of candidate genes (Kaltenbrun et al., 2011). Like the human heart, the *Xenopus* linear heart tube structure undergoes looping and multi-chamber formation, and demonstrates atrial and ventricular excitation wave patterns that are similar to His-Purkinje conduction in humans (Sedmera et al., 2003). Cardiac function is not required for embryo development; thus, molecular manipulation of the heart can be conducted *in vivo* (Mohun et al., 2000), including the termination of important signaling pathways. Like the zebrafish, 3D models of *Xenopus* cardiac stages can be constructed by using serial sectioning and microscopy imaging. *In vivo*, longitudinal 4D imaging can also be performed; for example, by using Doppler optical coherence tomography (Box 1), to study cardiac structure, conduction and blood flow, due to the large size and ease of manipulation of this model (Jenkins et al., 2012; Kaltenbrun et al., 2011; Mohun et al., 2000). MOs or transgenesis can both be used to investigate the roles of individual genes in normal cardiogenesis, including promoter–enhancer analyses, sequence mapping and the investigation of complex gene regulatory networks. However, the ability to perform gene targeting in *Xenopus* embryos to study cardiogenic proteins is hampered by low-level transgene expression and mosaicism in cardiogenic tissues that make dose-dependent effects difficult to appreciate, by insufficient sensitivity of standard *in situ* hybridization to detect low-level protein expression in cardiogenic tissues and the high mortality of gene-targeted embryos, as a high expression of transgenes can interfere with the normal expression of cardiogenic markers (Cleaver et al., 1996; Fu et al., 1998; Horb and Thomsen, 1999). Aberrant phenotypes may only occur with overexpression of transgenic proteins, thus restricting this translational utility of this model. Nonetheless, the *Xenopus* model has been informative for studying complex CHD (Table 2), its usefulness derived from elucidating the role of genes that are crucial in early cardiogenesis, i.e. the tinman homologs Nkx2-3 and Nkx2-5 9 (Box 1), and in causation of septal defects, i.e. transcription factor Tbx5, and heterotaxy syndrome (see Box 1), i.e. Zinc finger of the cerebellum protein Zic3 (Fu et al., 1998; Horb and Thomsen, 1999; Kitaguchi et al., 2000).

### Fly

*Drosophila melanogaster* is an extremely versatile and genetically tractable model, with readily available molecular tools that can be used to gain new insights into cardiogenesis and heart physiology (Camacho et al., 2016; Rotstein and Paululat, 2016; Vogler and Bodmer, 2015). Unlike the closed circulatory system that comes with high hydrostatic pressure in humans, the developing *Drosophila* heart is a simple structure, comprising a heart tube that pumps haemolymph in an open circulatory system at low hydrostatic pressure and starts beating upon completion of embryogenesis (Rotstein and Paululat, 2016). This simple, *in vivo* model provides the means to evaluate evolutionarily conserved genetic control mechanisms and developmental processes (Bier and Bodmer, 2004). There is general functional conservation of genes, cardiac proteins, ECM components (e.g. collagens, laminins and integrins) and cardiac precursors between flies and mammals (Bryantsev and Cripps, 2009; Hughes and Jacobs, 2017; Kooij et al., 2014; Reim and Frasch, 2005). Indeed, ∼30% of human disease-causing genes with homologs in *Drosophila* have the same function in humans and flies (Bier and Bodmer, 2004; Reiter et al., 2001). Tools have also been developed to perform large-scale genetic screens to identify disease-causing mutations, such as the novel overexpression activity (NOVA) screen (Guichard et al., 2002). Because the *Drosophila* embryo does not rely on the circulatory function of the heart during development, physical, cellular and subcellular manipulations to its cardiac function can be performed during embryogenesis to assess their downstream effects *in vivo* (Sénatore et al., 2010), a feat that cannot be achieved in higher-order vertebrates. This has advanced our understanding of the crucial roles that certain signaling pathways play in cardiogenesis, including the function of the Slit-Robo complex (Box 1), ECM components involved in cardiomyocyte contractility (Nishimura et al., 2014; Vogler and Bodmer, 2015) and of the highly conserved homeobox protein Nkx2-5 (Tanaka et al., 2001). Mutations in genes that encode components of such pathways are associated with septal and valvular defects, and with abnormal venous connections to the heart, including TOF and atrioventricular conduction delays (Bartlett et al., 2007; Schott et al., 1998). Live imaging of *Drosophila* cardiac development can also be enhanced by using fluorescent reporter transgenes (Morin et al., 2001). Thus, the simple genetic and cardiac system of *Drosophila* is an advantage when attempting to comprehend the multi-gene interactions that contribute to CHD. Limited by its simple structure, *Drosophila* cannot be used for observing morphological defects that arise from cardiac looping, septation or conduction events (Bier and Bodmer, 2004).

### Chick

In the chick (*Gallus gallus domesticus*), cardiac progenitors, migrating cardiac neural crest cells and a proepicardium (Box 1) develop to give rise to a primary heart tube, which then undergoes looping to form chambers, outflow tracks and a conduction system (Martinsen, 2005). In addition to the analysis of genetic mutations, chick embryos are useful for studying the mechanobiology of the CVS and the consequences of hemodynamic perturbations, as they are amenable to simple surgical manipulations, including cardiac neural crest ablation and outflow track obstruction; they can also be imaged *in ovo* by using optical coherence tomography (Midgett et al., 2017). Our current understanding of how faulty cardiac neural crest cell migration and abnormal endocardial cushion development contribute to cardiac outflow tract malformations, aorta and pulmonary trunk mispositioning, and myocardial dysfunction, derive from studies in the chick (Hutson and Kirby, 2007; Kirby et al., 1985, 1983; Yelbuz et al., 2002). Models of outflow track obstruction created by ligation of the left pulmonary artery or the left atrial appendage in the chick have been used to elucidate the relationship between hemodynamic flow, vessel wall shear stress, chamber and valve formation and myocardial function, as summarized in Table 2 (Johnson Kameny et al., 2019; Tobita and Keller, 2000). The chick also lends itself to the study of signaling pathways involved in cardiogenesis, being more readily genetically manipulable than mice. Mutations studied in the chick embryo are summarized in Table 2. In terms of genome manipulation, the chick is lagging behind the mouse due to technical difficulties with introducing tools for gain- or loss-of-function (Gammill and Krull, 2011; Sauka-Spengler and Barembaum, 2008). The recent technical ability to transiently knockdown endogenous protein, primarily by using short-hairpin RNAs (shRNAs) expressed from plasmids to interfere with RNA expression (continuous shRNAs production, inexpensive) or antisense MO (effect diluted with cell divisions, expensive), both introduced by electroporation, offers expanded opportunities to effectively study functions of specific genes (Dai et al., 2005; Eisen and Smith, 2008). Although not as genetically tractable as the mouse – in terms of simulating syndromic CHD – the chick is still a valuable model for structural cardiac diseases (Suzuki et al., 2009; Tutarel et al., 2005); however, it is important to note that certain cardiac events differ between chicks and humans, including the development of the septum secundium and the pharyngeal arch artery system. As such, it might not always be possible to faithfully recapitulate in chicks the abnormal cardiogenesis present in human CHD patients (Martinsen, 2005).

### Mouse

In the mouse (*Mus musculus*), the heart begins as a linear tube, which then loops and forms a four-chambered structure, and then undergoes further development, including septation and vessel formation (Andersen et al., 2014; Moorman and Christoffels, 2003). Numerous human disease models have been generated in mice by introducing or disrupting genes of interest in mouse embryos. In the case of CHD, the resulting abnormal phenotypes have provided many new insights into the molecular mechanisms of human cardiac disease, as summarized in Table 2 (Kloesel et al., 2016). The significant anatomical and developmental similarities that exist between mice and humans, i.e. cardiac looping, valvuloseptal formation, myocardial compaction and trabeculation (Desgrange et al., 2018; Ivanovitch et al., 2017; Le Garrec et al., 2013; Zaffran et al., 2004), allow clinically relevant data to be derived from some mouse models (Wessels and Sedmera, 2003). In addition, genetic knockout mouse models of cardiac neural crest mutations recapitulate the phenotypes of physical neural crest ablation in the chick (Hutson and Kirby, 2003, 2007).

Mice have been used to develop a number of CHD models, in which mechanistic pathways can be analyzed at cellular and molecular levels. The effective utilization of complementary advanced genetics, e.g. whole exome sequencing, bulk transcriptomics and scRNAseq, in transgenic mice that facilitate the study of morphological sequelae have enabled scientists to examine essential signaling and regulatory networks in cardiogenesis, uncovering stage-specific disturbances related to CHD (Li et al., 2014a,b). These powerful tools provide personalized models of genetic mutations that were identified in individuals with CHD, and help to understand gene functions and regulatory networks. Six significant examples are given in the following: (1) *Adamts19* knockout mice exhibit similar valvular disease as affected patients, thereby demonstrating its role in the perturbation of the WNT–ADAMTS19–KLF2 axis that is crucial for valvular development (Wünnemann et al., 2020). (2) The importance of semaphorin (Sema)/plexin signaling in cardiomyopathies was demonstrated by knocking down Plxnd1, a class-3 Sema receptor in endothelial cells, and caused excessive myocardial trabeculation and impaired compaction associated with Notch1 overexpression (Sandireddy et al., 2019). (3) *Cited2* mutations that perturb transcription factors and cardiopoietic gene expression in knockout mice, result in disrupted cardiac differentiation and septal defects (Santos et al., 2019). A defective transcription factor HAND1 in left ventricle cardiomyocytes, leads to disruption of regulatory pathways that control cardiac hypertrophy, cardiomyocyte proliferation, balanced development of cardiac cell lineages, septation, conduction and trabeculation (de Soysa et al., 2019; Firulli et al., 2020; Han et al., 2019). (5) Abberant endothelial-to-mesenchymal transition disrupts outflow tract formation (Liu et al., 2019). (6) Notch pathway disruption and abnormal cardiac cell behavior cause abnormal trabeculation, defective compaction and cardiomyopathy (Del Monte-Nieto et al., 2018).

Mice have also been used to assess the impact of environmental causes of CHD and to elucidate pathways of action at single-cell level. Examples include studies using teratogenic drugs, such as valproic acid, which is commonly used to treat epilepsy and inhibits histone deacetylases, thus, affecting transcription factors involved in cardiac structure development and cardiomyocyte differentiation, giving rise to myocardial disorganization and contractile dysfunction (Philbrook et al., 2019). Intrauterine hypoxia, e.g. during uteroplacental insufficiency that causes fetal growth restriction, reduces cellular proliferation in the second heart field due to downregulated FGF signaling, thereby causing global downregulation of protein synthesis of, for example, Nkx2-5 and hypoxia-inducible factor 1α, as well as perturbation of cardiac outflow tract formation, alignment or elongation, resulting in VSD, overriding aorta, double-outlet right ventricle (DORV) or transposition of great arteries (Box 1) (Moreau et al., 2019; Shi et al., 2016). Persistent hyperglycemia of gestational diabetes can lead to (i) embryopathy through enhanced production of reactive oxygen or reactive nitrogen species, thereby causing endothelial stress (Kumar et al., 2007, 2008; Moazzen et al., 2014; Wentzel et al., 2008); (ii) transcriptional alterations of genes crucial for cardiac development, e.g. *Vegfa*, *Bmp4*, sonic hedgehog homolog, thereby leading to increased apoptosis in the heart (Moazzen et al., 2014; Wentzel et al., 2008); (iii) inhibition of Wnt and Notch signaling (Wang et al., 2015); (iv) other cardinal transcriptional pathways, summarized in (Basu and Garg, 2018); (v) hyperglycemia-driven inhibition of cardiac stem cell differentiation and cardiomyocyte maturation (Nakano et al., 2017; Yang et al., 2016), and (vi) overstimulation of myocardial growth (Gordon et al., 2015).

Yet, mouse models are not without limitations. The murine CVS, for example, differs in important aspects from that of humans, i.e. regarding its substantially higher heart rate and distinct myocardial Na^+^/Ca^2+^ exchange mechanisms (Ottolia et al., 2013). Developing mouse knockout models is costly, as it requires assisted fertility and embryo manipulation. In addition, severe complex heart diseases are often perinatal lethal, leaving researchers with mice that express milder forms of disease (Bier and Bodmer, 2004). Despite these limitations, translational CHD research has benefited greatly from transgenic mice, and this model continues to be crucial in the search for molecular therapies to target aberrant genetic pathways.

### Sheep

The heart of sheep (*Ovis aries*) shares significant anatomical and physiological homologies with the human heart, providing a model with which to understand normal and pathological cardiogenesis at structural, cellular and molecular levels (Fishman et al., 1978; Weil et al., 1993). The size, gestation and physiology of the sheep fetus also enhances the value of this model, facilitating the study of *in utero* and perinatal effects of complex CHD (Johnson et al., 2014; Valdeomillos et al., 2019). CHD models in sheep are all surgically generated and used to investigate abnormal cardiac mechanobiology (summarized in Table 2). As twinning is common in sheep, this system naturally provides non-treated controls that can be studied in tandem.

Surgically generated models of CHD in sheep have, particularly, advanced our understanding of the developmental pathophysiology of outflow track obstruction. For example, they have revealed maladaptive changes that result in uncompensated right ventricular hypertrophy (RVH) and progress to heart failure (Montgomery et al., 1998), differences in miRNA expression profiles in compensated and uncompensated RVH, as well as the effects of failed repair (Johnson Kameny et al., 2019; Kameny et al., 2018; Van Puyvelde et al., 2019).

The main limitations of sheep as a species in which to model CHD is their lack of genetic tractability. Transgenic sheep would be prohibitively expensive to produce and, thus, to prove causation between candidate genes and observed morphology would be impossible. Nevertheless, surgical interventions can be trialed and tailored to the disorder studied, and sheep are valuable models to evaluate short- and long-term outcomes of intrauterine or early neonatal therapies (Schmidt et al., 2008). The sheep model also remains highly useful to study the mechanical sequelae of outflow tract obstruction, particularly because, as we cover in the next section, mechanobiology contributes greatly to our understanding of non-genetic non-syndromic CHD.

### In silico models of CHD

A recent novel approach to CHD research is the development of organ-level computational models to study the biomechanics and physiological functions of the prenatal CVS (Dewan et al., 2017; Trusty et al., 2018; Wiputra et al., 2018). This approach has been extrapolated from computational tools used to study adult CVS diseases (Meoli et al., 2015; Morris et al., 2016). The motivation for studying prenatal CVS biomechanics is based on evidence that cardiac biomechanics are crucial in the multifactorial aetiology of human CHD, highlighted in animal models (Table 2). For example, outflow obstruction that alters haemodynamic wall shear stresses in chick and zebrafish embryos leads to cardiac underdevelopment and left ventricular hypertrophy (LVH) (Hove et al., 2003; Tobita and Keller, 2000). These changes occur through altered expression of ET1, NOS3, KLF2 and PIEZO1, all of which are flow-responsive and mechanosensitive proteins, and crucially important for trabeculation and valve development (Duchemin et al., 2019; Faucherre et al., 2020; Groenendijk et al., 2007; Rasouli et al., 2018). Further, in *silent heart* (*sih*^−^^/−^) zebrafish embryos, which carry a *tnnt2* mutation, absence of mechanical stimuli from cardiac contractions prevented the formation of atrioventricular cardiac cushions (Bartman et al., 2004). Together, these data demonstrate conclusively the importance of biomechanics in cardiogenesis and the need to appreciate the mechanobiology of normal heart development.

The emergence of fetoscopy (Box 1) and ultrasound-guided interventions to treat CHD patients *in utero* demonstrates the potential benefit of prenatal biomechanical therapies to alter the course of cardiac morphological development, and mitigate the risks of structural abnormalities at birth (Freud et al., 2014; Gardiner, 2019; Tulzer et al., 2018). These interventions typically take place during the late-second to early-third trimesters, when a detailed 2D echocardiographic (2DE) study of structural abnormalities is feasible and when ultrasound-guided invasive procedures can be performed in anticipation of correcting the developmental path towards malformation. To give an example of such approach, in fetal critical aortic stenosis with evolving HLHS, the LV grows normally until aortic outflow obstruction occurs mid-gestation, causing abnormal ventricular pressures, contractile motions and, eventually HLHS – a potentially fatal condition that can only be treated by transplantation (Makikallio et al., 2006; Saraf et al., 2019). *In utero* aortic valvuloplasty (Box 1) can be performed in affected fetuses to relieve outflow obstruction, and has increased biventricular cardiac function at birth from 26% (without intervention) to 59% (Friedman et al., 2018; Makikallio et al., 2006).

**Fig. 4.**
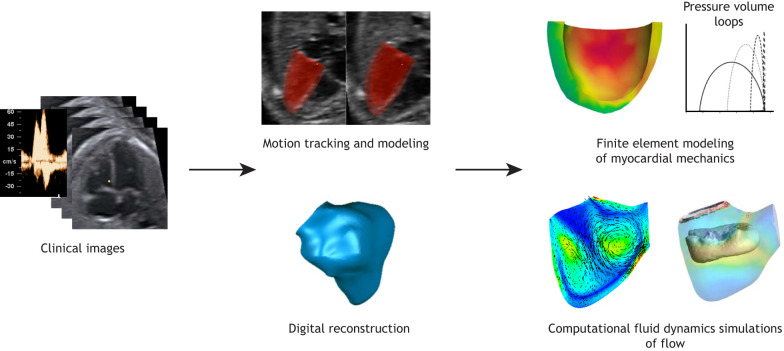
**Computational simulation of the human fetal heart.** Summary of the steps involved in *in-silico* modeling based on biomechanics derived from clinical ultrasound images. From left to right: Images are first processed to reconstruct the anatomy of the relevant cardiac structures. Motion tracking and modeling, as well as digital reconstruction can be carried out to accurately extract and mathematically model their motion. This is followed by finite element modeling of myocardial mechanics to understand myocardial stresses and strains, and to evaluate cardiac function under hypothetical conditions. Computational fluid dynamics can be simulated to reveal details of flow patterns and forces. Clinical and computational reconstruction images captured and developed by C.H.Y.

It is, thus, important to understand the biomechanical characteristics of the fetal heart, and their effect on cardiac morphological and functional development. A good way to achieve this is by using computational modeling, which can assess current biomechanical characteristics and predict the effects of any changes in developing cardiac structures. Computational models can also capture fetal heart growth and remodeling in response to biomechanical and haemodynamic alterations. These models can be broadly categorized into models of blood flow models (Courchaine and Rugonyi, 2018) and tissue mechanics (Dewan et al., 2017). Fig. 4 provides a schematic of the modeling process. Blood flow modeling is performed by using computational fluid dynamics (CFD) simulations, which involve computational reconstructions of the blood volume of interest, dividing it into many small elements (meshing), followed by iteratively solving the Navier–Stokes governing equations (Box 1) of fluid motion for each of these elements, in order to derive a description of flow and flow forces within the volume (Bluestein, 2017). CFD modeling of the human fetal heart has been performed in normal healthy environments, and in congenital heart malformations, and is explained as follows. 4D clinical ultrasound fetal heart images were obtained using spatio–temporal image correlation (STIC; Box 1) from structurally normal human fetuses between 20–32 weeks of gestation. These images were used to extract data on ventricular anatomy and ventricular wall motion, which were then used to generate fluid dynamic simulations (Lai et al., 2016; Wiputra et al., 2016). Collectively, Lai et al., and Wiputra et al. described the same flow patterns for the left and right ventricles – a pair of vortex rings emanating from the mitral or tricupsid inlet during diastole, corresponding to E- and A-waves (Box 1) – the primary mechanisms by which flow shear stresses are imposed on the endocardial wall. The fetal right ventricle (RV) has been reported to contract with a wave-like motion from the sinus to the infundibulum; computational modeling indicates that this is to conserve energy required for blood ejection (Wiputra et al., 2017). The same approach was used to model the flow in hearts of fetuses with TOF between 21 and 32 weeks of gestation (Wiputra et al., 2018). Wiputra and colleagues observed that TOF hearts need to work harder to eject blood against the outlet obstruction (pulmonary valve stenosis), and the consequential high-volume flow through the RV caused higher wall shear stresses and chaotic flow patterns. In these fetal hearts, VSD – which is part of TOF – created a connection between both ventricles to equilibrate their pressures and to prevent higher pressures in either ventricle (Wiputra et al., 2018). These alterations are considered influential on the developing morphology of the fetal heart.

Computational modeling of blood flow dynamics in fetal blood vessels have also been conducted to better understand flow patterns and forces in fetal coarctation of the aorta (CoA; Box 1) (Chen et al., 2017). 3D STIC fetal echocardiography images of the aortic and ductal arches of normal human fetuses at 32 weeks of gestation were obtained and computational models with reduced aortic isthmus (Box 1) dimensions created to emulate CoA. Chen and colleagues demonstrated that increased narrowing of the isthmus modifies vortical and helical flow patterns, causing substantial increase in velocity and wall shear stresses, and reduction in descending aortic pressure. Given that prenatal diagnosis of CoA can be challenging (Buyens et al., 2012; Familiari et al., 2017), such computational modeling may prove useful to enhance diagnostic accuracy of related haemodynamic changes, which, in turn, may influence patient morbidity and survival (Franklin et al., 2002). In another study, computational modeling of blood flow in the ductus venosus (DV; Box 1) in chromosomally or structurally abnormal fetuses was performed using 3D computational simulation and Doppler measurements, and alterations in the characteristic DV flow patterns and pressures were observed (Rezaee and Hassani, 2016).

In addition to 3D flow simulations, fetal circulation has also been modeled using a one-dimensional approach, known as Windkessel or lumped-parameter modeling (Shi et al., 2011). In this approach, a network of electronic circuitry is used as an analogue for vascular flow resistance and compliances. This approach removes the complexity of modeling any one organ, allowing the entire circulatory system to be included in the model, which is useful for testing systemic responses to pathophysiological changes in CHD. This approach has been used to model fetal circulation in pregnancies complicated by intrauterine growth restrictions, in order to understand reactive changes to cerebral and placental vascular resistance (Garcia-Canadilla et al., 2014; van den Wijngaard et al., 2006). Pennati et al. have also developed a carefully considered Windkessel fetal circulation model based on *in vivo* measurements, and have recommended scaling factors that can be applied to this model at various gestational ages (Pennati and Fumero, 2000).

Tissue mechanics modeling of the fetal CVS has been achieved mainly in the form of finite element modeling (FEM) of myocardial biomechanics. The FEM method divides the solid structure of interest into many small elements, and iteratively solves the governing equations of momentum and deformational mechanics within these elements, to derive a prediction of the deformational and stress characteristics of the object of interest (Lluch et al., 2019). The technique has originally been developed by civil and aeronautical engineers to study structural stability and aircraft structural integrity respectively, and was later imported into biomedical engineering to study myocardial and vascular biomechanics (Costopoulos et al., 2017; Shavik et al., 2018). In one FEM modeling study of the human fetal heart, anatomic dimensions were extracted from 2DE studies of normal fetuses between 20 and 37 weeks of gestation. Myocardial stiffness and active contractions have been mathematically described with an anisotropic hyperelastic constitutive law (Box 1), and myofiber orientations measured from published histology were adopted in the model (Peña et al., 2010). The authors concluded that fetal myocardial active tension increases significantly between 20 and 37 weeks of gestation, which was attributed to myocyte enlargement, differentiation and proliferation. A recent FEM modeling study of human fetal hearts between 22 and 32 weeks of gestation provided insights into the pathophysiology of fetal aortic stenosis with evolving HLHS (Ong et al., 2020). The study calculated the pressures, myocardial strain and stresses, and blood flow velocities at the heart valves at various degrees of stenosis severity, endocardial fibroelastosis, LV wall thickening and loss of contractility. The authors found that changes to myocardial stiffness due to fibroelastosis do not impede cardiac function. By using clinical measurements as comparisons, they further concluded that LV hypertrophy and reduced contractility are likely to manifest after aortic stenosis. FEM modeling has also demonstrated the ability to model the morphological growth of normal and HLHS fetal hearts. These studies accurately replicated the published measurements of left ventricular volumes at progressive gestational ages for both normal and diseased groups, reporting that reduced ventricular filling, chamber shape deformation and myocardial stresses influence the morphological alterations that result in HLHS (Dewan et al., 2017; Ohayon et al., 2002).

These computational modeling techniques have found much success when applied to investigating postnatal and adult-onset disease, designing treatments, and assisting clinical decision-making. For example, in patients with single-ventricle defects scheduled for Fontan surgery (Box 1), computational simulations of blood flow have been used to assess preoperative flow conditions and to predict the outcomes of various surgical options (Trusty et al., 2018). Fluid-structure interaction simulations have also been deployed to evaluate transcatheter aortic valve replacement, to identify potentials to optimize device design (Wu et al., 2016), whereas FEM simulations have been used to evaluate the effectiveness of novel procedures, such as leaflet laceration after valve-in-valve procedures (Yaakobovich et al., 2020). Application of the same techniques to fetal CVS and CHD research can have similar translational potential. For example, computational modeling to predict the outcomes of intrauterine surgical interventions to treat CHD may enhance patient selection and intervention planning, to minimize risks and to maximize the potential benefits.

### Understanding epigenetic contributions to CHD

Despite the numerous CHD syndromes caused by single-gene variants (Table 1), specific causal mutations are recognized in only 20–30% of sporadic CHD (Mitchell et al., 2007; Pierpont et al., 2007), as revealed by whole-exome and -genome sequencing efforts (Homsy et al., 2015; Jin et al., 2017; Zaidi et al., 2013). Gene mutations account for only 35% of CHD. Importantly, many of these genes encode epigenetic pathway components, such as histone-modifying enzymes, leading to investigations of molecular epigenetics or pathways that regulate gene expression programs in CHD. Non-coding genomic regulatory loci, marked by epigenetic profiles and deregulated gene expression programs, are also likely to play important roles in the pathophysiology of CHD and its sequelae (Jarrell et al., 2019). Genetic variants at such non-coding regulatory sequences might influence the presentation of complex disorders by influencing chromatin accessibility, histone modifications, and downstream consequences of chromatin remodeling and gene expression (Table 3).

**Table 3.**
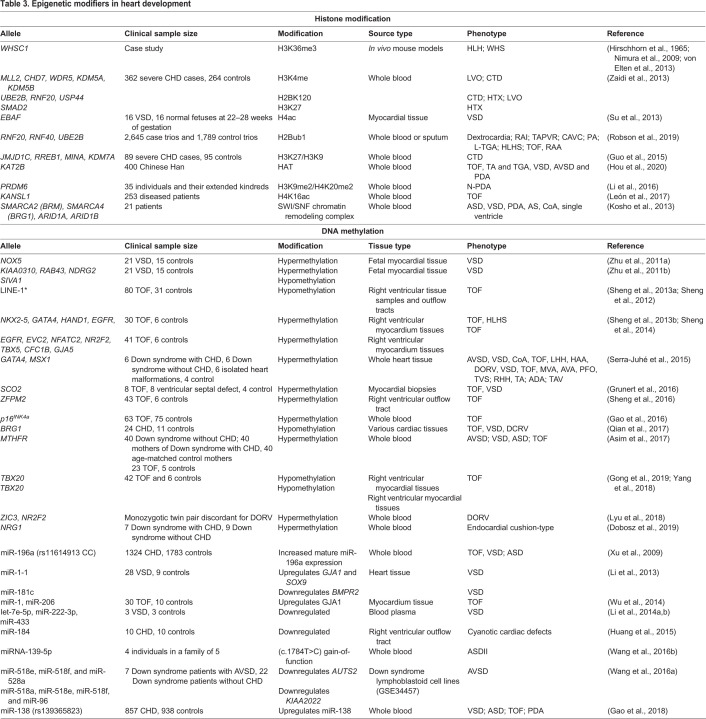
**Epigenetic modifiers in heart development**

Cardiac epigenomic maps have been generated for human and rodent hearts (Preissl et al., 2015; VanOudenhove et al., 2020), but only little is known about how specific histone modifications contribute to individual CHD phenotypes. Our understanding of epigenetic enhancer-promoter regulation of cardiogenesis has recently advanced with the development of genome-wide association studies (GWAS) and chromatin quantitative trait loci (chQTL; Box 1) analyses, elucidated by the epigenetic profiling of specific tissues, and by chromosome conformation capture (3C) and its derivatives (4C, Hi-C and HiChIP) that profile the spatial interaction of loci within and between chromosomes. Epigenetic factors contribute to phenotypic variation and are valuable for prioritizing variants for disease association (Kirk et al., 2006; Koopmann et al., 2014; Sati and Cavalli, 2017). For example, GWAS have associated non-coding, single nucleotide polymorphisms (SNPs) with various forms of CHD, although understanding the molecular functions of these intergenic SNPs remains quite challenging (Theis et al., 2015; Wijnands et al., 2017). One study has reported that a non-coding enhancer SNP disrupts binding of the transcription factor Tfap2a in the zebrafish heart, perturbing the expression of the atrial fibrillation-associated gene *pitx2c* through long-range interaction (Ye et al., 2016). Mutations in and the deletion of genes that encode histone modifying enzymes (e.g. EZH2, EP300) also produce deleterious effects on cardiovascular development in mice (Chen et al., 2009, 2012; He et al., 2012). However, to identify the disease regulatory variants with GWAS alone remains a challenge owing to the large cohort sizes needed to achieve statistical power, to disease heterogeneity and the need to assign *cis*-regulatory functions for implicated candidate loci (Ferrero, 2018). Other challenges in assessing epigenomic influences include modifier genes that are difficult to identify and characterize, candidate genes or sequence variants that are associated with a variety of heart malformations, and genes and variants that display incomplete penetrance, and may even be found in normal phenotypes (Farr et al., 2018).

## Conclusions

A key aim of CHD research is to develop personalized therapies for prenatally diagnosed CHD, which can correct a known genetic mutation through gene-editing or gene addition, or that can correct abnormal biomechanics through minimally invasive surgical techniques. The fetal approach is ideal because it enables clinicians to arrest abnormal cardiac development before end-organ damage can occur, thereby producing better clinical outcomes (Kumar and Lodge, 2019).

A key challenge remains the further development of safe and effective therapies by using clinically relevant, large animal models. These efforts are supported by advances in gene modification technologies, which should enable large animal models to be genetically engineered such they carry common genetic mutations identified in CHD patients. We expect these technologies to be developed in tandem with molecular therapies to target the at-risk fetus *in utero*, aiming to correct cardiogenesis as early as possible in order to arrest pathology before it results in ventricular hypertrophy, hypotrophy or heart failure. Additionally, correcting CHD should be considered through ethical lenses, so that a potential novel treatment can be assessed not only on its ability to tackle a scientific challenge and rectify the disease, but also on its benefits to the patient and avoidance of harm.
